# Association of serum high creatinine-to-albumin ratio with increased 1-year mortality in older patients following hip fracture surgery

**DOI:** 10.3389/fmed.2026.1730691

**Published:** 2026-02-09

**Authors:** Peipei Jin, Jian Huang, Wei Yuan, Housheng Wang, Wubin Chen, Ling Chen, Wenbin Lu

**Affiliations:** 1 Faculty of Anesthesiology, Changhai Hospital, Naval Military Medical University, Shanghai, China; 2 Department of Anesthesiology, Shanghai 411 Hospital, Shanghai University, Shanghai, China; 3 Department of Anesthesiology, Shuguang Hospital Affiliated with Shanghai University of Traditional Chinese Medicine, Shanghai, China

**Keywords:** aged, creatinine-to-albumin ratio, hip fracture, mortality, surgery

## Abstract

**Background:**

Creatinine-to-albumin ratio (CAR) was associated with short-term and long-term prognosis in various clinical settings. However, the relation of CAR with prognosis in older patients with hip fracture remains unclear.

**Methods:**

This retrospective study was performed in older patients undergoing hip fracture surgery in our hospital from January 2018 to December 2023. Serum creatinine and albumin were recorded at admission. The primary outcome was 1-year mortality after surgery in patients with hip fracture. The association between preoperative serum creatinine-to-albumin ratio and 1-year mortality was assessed by Kaplan–Meier curves, Cox regression models, and stratified analyses.

**Results:**

The mean age of the participants was 79.5 ± 8.1 years and the median CAR was 1.9 (1.5–2.4). The prevalence of 1-year mortality following hip fracture surgery was 8.6%. Multivariate Cox regression analysis indicated that high CAR was independently associated with increased 1-year mortality (hazard ratio = 1.49; 95% confidence interval = 1.02–2.17) after adjusting for covariates. In addition, Kaplan–Meier curve analyses indicated that patients with the high CAR had a higher 1-year mortality than that with low CAR (*p* < 0.0001). Subgroup analyses showed that the association was similar across all subgroups.

**Conclusion:**

The CAR can serve as an independent prognosis indicator for 1-year mortality in older patients following hip fracture surgery. However, researchers are required to confirm the findings in the future.

## 1 Introduction

With the acceleration of global population aging, the incidence of hip fractures in the older individuals is on the rise, becoming a significant public health issue (1, 2). Hip fractures are one of the most common injuries in the older patients, with poor prognosis, high complication rates, and a 1-year mortality rate of up to 15–25% (3, 4), which put unprecedented demands on healthcare infrastructure. In this context, identifying factors associated with postoperative mortality is critical to improve risk stratification and guide personalized treatment strategies.

Previous studies have shown that inflammation, malnutrition, and renal function impairment were related with poor prognosis after surgery (5–7). Accumulating evidence has shown that creatinine-to-albumin ratio (CAR), a biomarker that integrates renal function, inflammation and nutrition, was associated with short-term and long-term prognosis in patients with acute coronary syndrome, diabetes and inflammatory diseases (8–10). In addition, recently, a retrospective cohort study indicated that increased CAR can serve as a risk factor for 28-day mortality in patients undergoing cardiac surgery (11). Furthermore, a prospective cohort study showed that CAR was related to 1-year mortality after surgery in patients with non-ST-elevation acute coronary syndrome (12). However, the association between CAR and mortality in older patients following hip fracture surgery has not been investigated.

Therefore, we hypothesized that serum CAR levels were associated with 1-year mortality after surgery in older patients with hip fractures. Our study provided a new insight to identify risk factors for mortality after surgery and optimize personalized care pathways for older patients with hip fractures.

## 2 Materials and methods

### 2.1 Study design and population

This retrospective observational study was performed in patients undergoing hip fracture surgery in our hospital from January 2018 to December 2023. This study was conducted in line with the Declaration of Helsinki and was approved by the ethical review committee in our hospital (CHEC2024-257). Due to the retrospective collection of de-identified clinical data from patients, informed consent was waived by the ethical review committee in our hospital.

Older adults with hip fracture (including femoral neck fractures and intertrochanteric fractures) who underwent surgery were eligible for study enrollment. Exclusion criteria included multiple fractures, renal function impairment, dementia, and age <65 years old. We also excluded patients with missing values on creatinine or albumin. Renal function impairment was defined according to an estimated glomerular filtration rate <60 mL/(min × 1.73 m^2^) using the Chinese-based equation (13).

### 2.2 Variables

We selected the variables based on existing literature and clinical practice (14, 15). Data regarding age, gender, body mass index (BMI), American Society of Anesthesiologists (ASA) physical status classification, type of anesthesia, alcohol history, smoking history, time to admission (time from injury to admission) and medical history were recorded in this study. Medical history included hypertension, diabetes, cardiovascular disease, cerebrovascular disease, lung disease, and cancer. In addition, creatinine, albumin, lactate dehydrogenase (LDH), glucose, D-dimer, activated partial thromboplastin time (APTT), international normalized ratio (INR), hemoglobin, lymphocyte, monocyte, neutrophil, and platelet were recorded at admission. The CAR was calculated as preoperative creatinine (μmol/L)/albumin (g/L). We employed multiple imputation to address variables with missing values <10%, with exception of CAR.

### 2.3 Outcome

The primary endpoint was 1-year mortality after surgery in older patients with hip fracture.

### 2.4 Statistical analysis

Participants were divided into two groups based on their survival status. Continuous variables with normally distributed data were presented as mean ± SD and were compared using Student’s *t*-test. Mann–Whitney *U*-test was used to compare continuous variables in non-normal distribution data described as medians (quartiles). Categorical variables were presented as frequencies (%) and analyzed using *χ*^2^ test.

The CAR levels were categorized as low CAR (<1.9) or high CAR (≥1.9), based on the median. Kaplan–Meier survival curves with log-rank testing were constructed to compare cumulative survival probabilities across low and high CAR groups. Dose–response relationship between CAR and 1-year mortality was assessed by the restricted cubic splines. We used multivariate Cox proportional hazard models to evaluate the association of CAR with 1-year mortality after surgery when CAR were both continuous and binary variables.

Variates with a *p*-value of <0.05 in the comparison of baseline characteristics between survival and death group were incorporated into multivariate models for covariate adjustment. In Model I, no covariates were adjusted. Model II was adjusted for age, gender, and BMI. Model III was adjusted for model II plus ASA physical status, time to admission, cerebrovascular disease, lung disease and cancer. In the model IV, LDH, INR, hemoglobin, lymphocyte count and neutrophil count were further adjusted.

Stratified and interaction analyses were performed, considering age (<80 years or ≥80 years), gender (male or female), ASA physical status (<III or ≥III), and the presence or absence of hypertension, diabetes, and cardiovascular disease. For each stratification, adjustments were made for potential confounders, including age, gender, BMI, ASA physical status, time to admission, cerebrovascular disease, lung disease, cancer, LDH, INR, hemoglobin, lymphocyte count, and neutrophil count, with exception of the stratification factor itself. The R software packages (The R Foundation)^1^ and Free Statistics software versions 1.7.1 were used to perform all statistical analyses. A *p*-value of less than 0.05 was considered to indicate statistical significance.

## 3 Results

**Figure 1:**
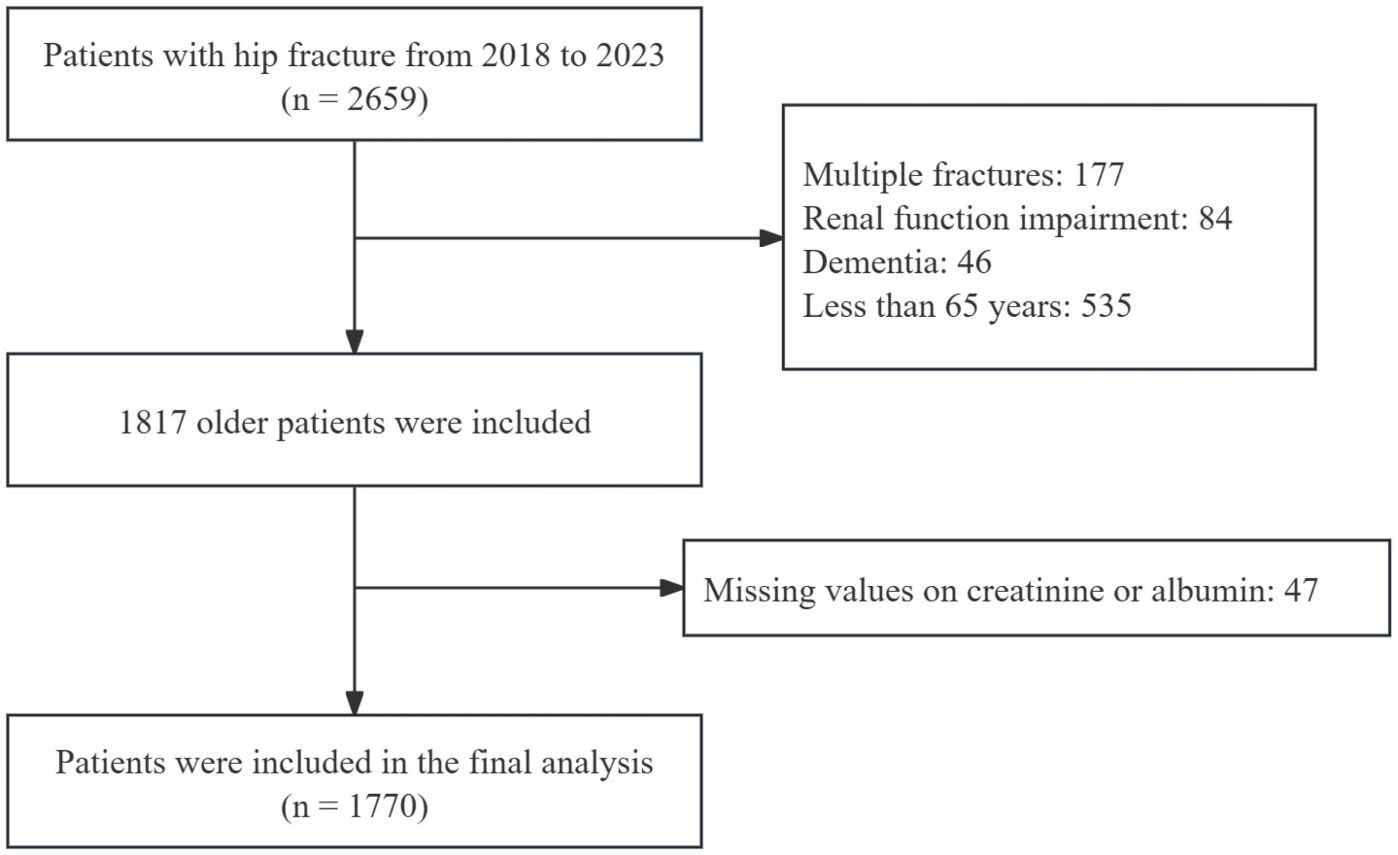
Flowchart of the study population selection process.

In total, 2,659 patients underwent hip fracture surgery were identified. After excluding 889 patients (177 had multiple fractures, 84 had renal function impairment, 46 had dementia, 535 were under the age of 65 years, and 47 had missing data on creatinine or albumin), the final data analysis included 1770 patients. The participant flow diagram is shown in Figure 1.

### 3.1 Participants characteristics

Table 1 shows the baseline information for all patients. The mean age of the study patients was 79.4 ± 8.1 years. The median baseline CAR was 1.9 (1.5–2.4); 1-year mortality rate after hip fractures surgery was 8.6% in older patients. Compared with survival group, patients in death group were older, predominantly male, had lower BMI, higher ASA physical status, and longer time from injury to admission (all *p* < 0.05). Additionally, patients with cerebrovascular disease, lung disease or cancer were more likely to experience 1-year mortality. In terms of laboratory parameters, patients in the death group exhibited lower levels of albumin, hemoglobin, and lymphocytes, while having higher levels of creatinine, CAR, LDH, INR, and neutrophils compared with those in the survival group (all *p* < 0.05).

**Table 1:** Demographics and clinical characteristics between two groups.

Variables	Total (*n* = 1770)	Survival group (*n* = 1,617)	Death group (*n* = 153)	*p*
Age (years)	79.4 ± 8.1	78.8 ± 7.9	85.5 ± 7.5	<0.001
Gender				0.028
Male	555 (31.4)	495 (30.6)	60 (39.2)	
Female	1,215 (68.6)	1,122 (69.4)	93 (60.8)	
BMI (kg/m^2^)	22.4 ± 3.7	22.5 ± 3.7	21.4 ± 3.8	<0.001
ASA physical status				<0.001
<III	1,068 (60.3)	999 (61.8)	69 (45.1)	
≥III	702 (39.7)	618 (38.2)	84 (54.9)	
Anesthesia				0.558
General anesthesia	283 (16.0)	256 (15.8)	27 (17.6)	
Spinal anesthesia	1,487 (84.0)	1,361 (84.2)	126 (82.4)	
Type of fracture				0.266
Femoral neck fractures	955 (54.0)	879 (54.4)	76 (49.7)	
Intertrochanteric fractures	815 (46.0)	738 (45.6)	77 (50.3)	
Type of surgery				0.288
Total hip arthroplasty	214 (12.1)	202 (12.5)	12 (7.8)	
Hemiarthroplasty	546 (30.8)	496 (30.7)	50 (32.7)	
Proximal femoral nail	770 (43.5)	697 (43.1)	73 (47.7)	
Open reduction and internal fixation	240 (13.6)	222 (13.7)	18 (11.8)	
Alcohol history	29 (1.6)	27 (1.7)	2 (1.3)	1
Smoking history	62 (3.5)	60 (3.7)	2 (1.3)	0.122
Hypertension	973 (55.0)	889 (55)	84 (54.9)	0.986
Diabetes	493 (27.9)	446 (27.6)	47 (30.7)	0.408
Cardiovascular disease	406 (22.9)	364 (22.5)	42 (27.5)	0.165
Cerebrovascular disease	272 (15.4)	237 (14.7)	35 (22.9)	0.007
Lung disease	156 (8.8)	127 (7.9)	29 (19)	<0.001
Cancer	49 (2.8)	40 (2.5)	9 (5.9)	0.033
Time to admission (h)	20.0 (6.5–48.0)	19.5 (6.0–48.0)	24.0 (10.0–72.0)	0.007
Creatinine (μmol/L)	66.0 (56.0–82.0)	66.0 (56.0–81.0)	71.0 (60.0–90.0)	0.002
Albumin (g/L)	36.0 (33.0–39.0)	36.0 (33.0–39.0)	33.0 (30.0–35.0)	<0.001
CAR	1.9 (1.5–2.4)	1.8 (1.5–2.3)	2.2 (1.8–2.9)	<0.001
LDH (U/L)	221.0 (190.0–257.0)	220.0 (190.0–256.0)	228.0 (197.0–274.0)	0.041
Glucose (mmol/L)	6.7 (5.7–8.4)	6.7 (5.7–8.3)	6.7 (5.8–9.6)	0.283
D-dimer (mg/L)	3.7 (1.8–8.3)	3.6 (1.7–8.4)	3.9 (2.5–7.5)	0.166
APTT (s)	36.8 (33.3–40.5)	36.8 (33.4–40.3)	36.5 (33.2–42.0)	0.480
INR	1.1 (1.0–1.1)	1.1 (1.0–1.1)	1.1 (1.1–1.2)	<0.001
Hemoglobin (g/L)	115.0 (101.0–128.0)	115.0 (102.0–129.0)	103.0 (89.0–123.0)	<0.001
Lymphocytes (10^9^/L)	1.0 (0.8–1.4)	1.1 (0.8–1.4)	0.9 (0.6–1.2)	<0.001
Monocytes (10^9^/L)	0.6 (0.4–0.8)	0.6 (0.4–0.8)	0.6 (0.4–0.8)	0.432
Neutrophils (10^9^/L)	6.4 (4.9–8.1)	6.3 (4.9–8.0)	7.1 (5.3–9.2)	<0.001
Platelets (10^9^/L)	177.0 (138.0–218.0)	177.0 (138.0–218.0)	186.0 (150.0–224.0)	0.061

Data are presented as mean ± SD or median (quartiles) and frequency (%). BMI, body mass index; ASA, American Society of Anesthesiologists; CAR, creatinine-to-albumin ratio; LDH, lactate dehydrogenase; APTT, activated partial thromboplastin time; INR, international normalized ratio.

### 3.2 Relationship between CAR and mortality after surgery

**Figure 2:**
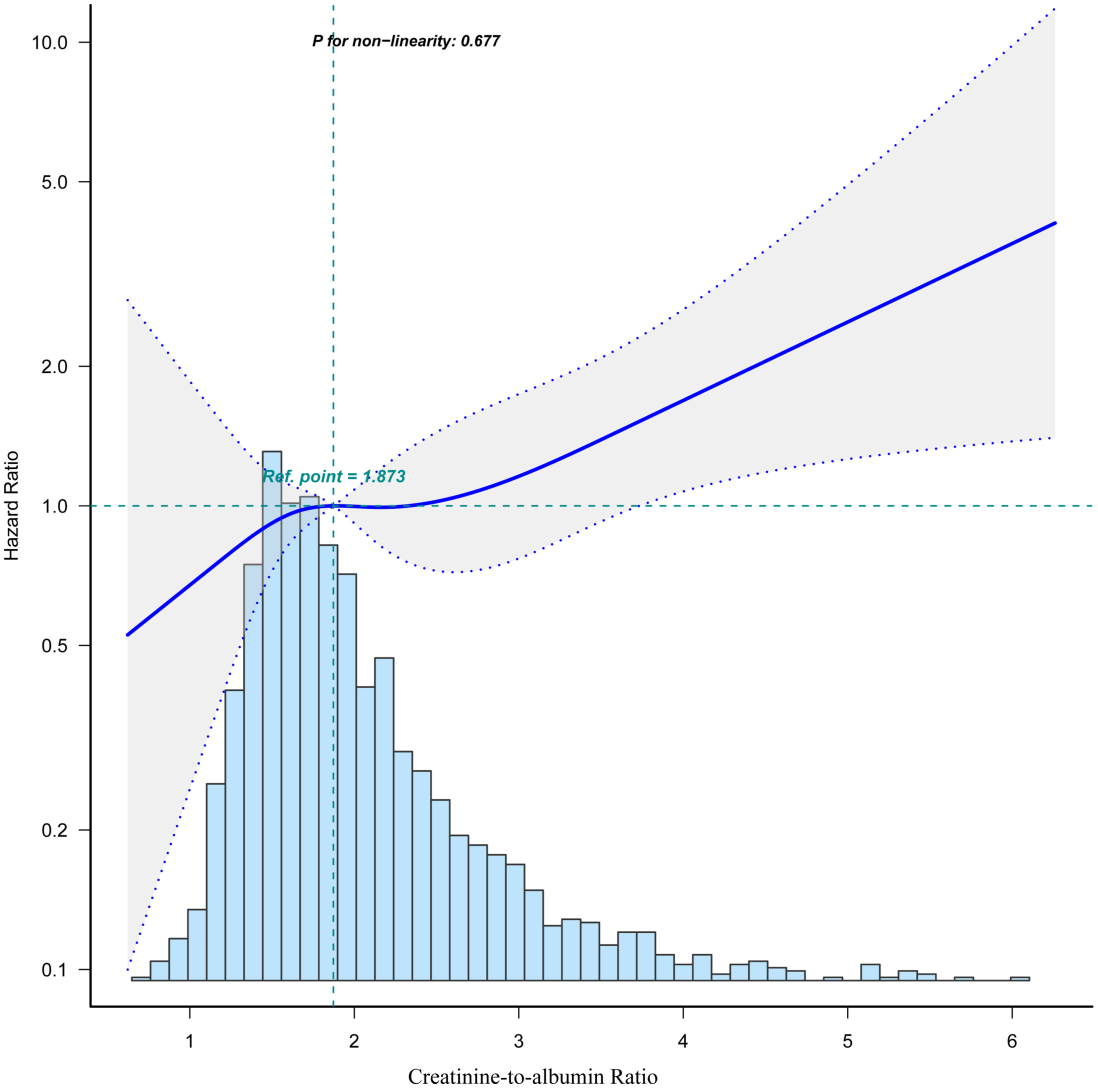
Dose–response relationship between CAR and 1-year mortality following hip fracture surgery by smooth curve fitting. Data were fit by a Cox proportional hazard regression model based on restricted cubic splines. CAR was entered as continuous variable. Data were adjusted for the factors (age, gender, BMI, ASA physical status, time to admission, cerebrovascular disease, lung disease, cancer, LDH, INR, hemoglobin, lymphocyte count and neutrophil count). The curves line and shaded areas around depict the estimated values and their corresponding 95% confidence intervals. The histogram depicts the distribution of the CAR. BMI, body mass index; ASA, American Society of Anesthesiologists; LDH, lactate dehydrogenase; INR, international normalized ratio; CAR, creatinine-to-albumin ratio.

**Figure 3:**
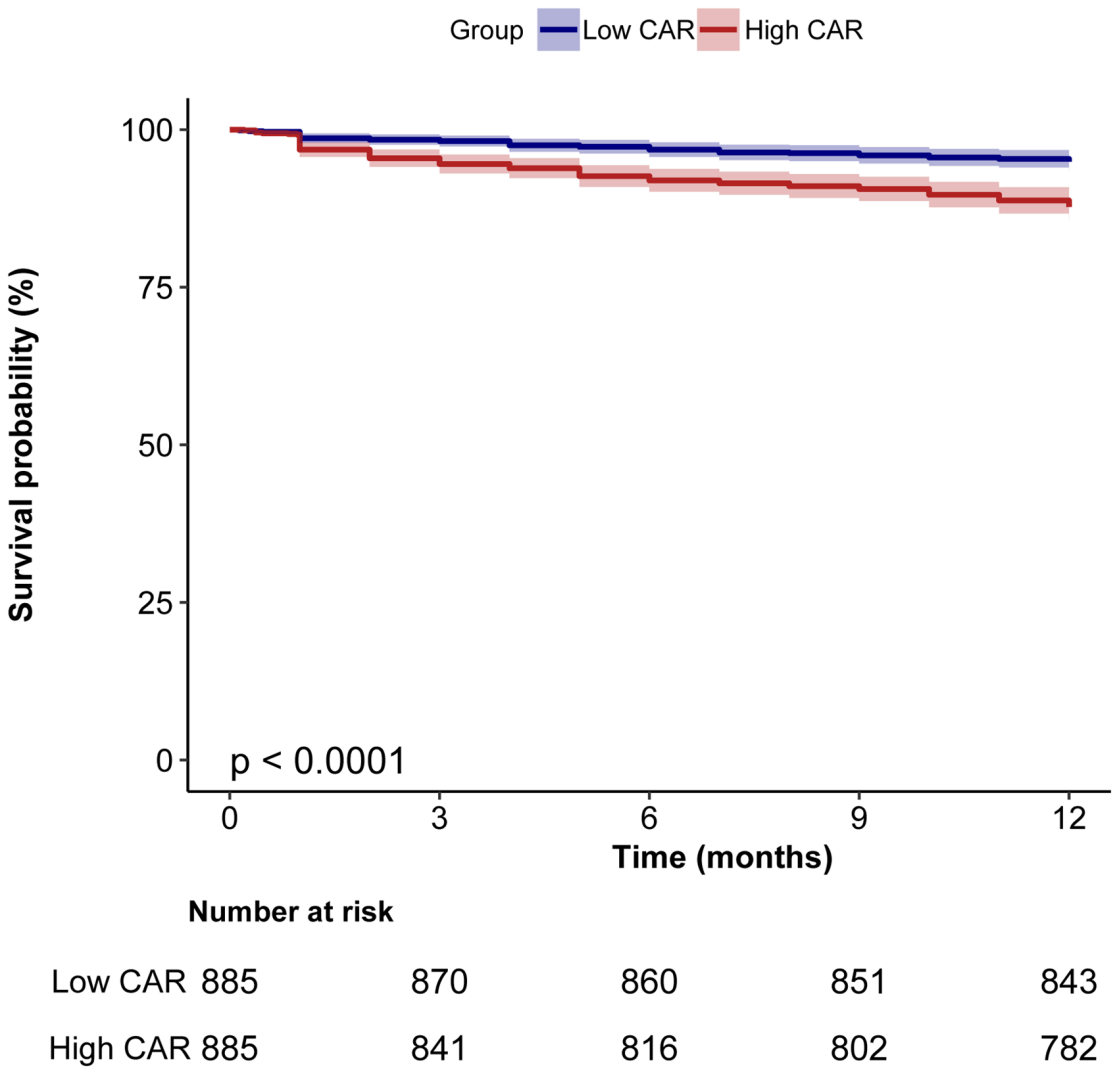
Kaplan–Meier survival curves of 1-year mortality for patients with hip fracture surgery.

Figure 2 shows the linear relationship between CAR and 1-year mortality after surgery in older patients with hip fracture. Kaplan–Meier survival analysis further revealed that patients in the low CAR group had better survival probabilities compared to those in the high CAR group (*p* < 0.0001; Figure 3). Moreover, multivariate Cox regression analysis showed that a high CAR was independently related to increased 1-year mortality regardless of continuous variable [hazard ratio (HR) = 1.30, 95% confidence interval (CI) = 1.07–1.58] or categorical variable [HR = 1.49, 95% CI = 1.02–2.17] in the adjusted model (Table 2).

**Table 2:** Univariate and multivariate Cox regression analysis to assess the association between CAR and 1-year mortality in the older patients with hip fracture surgery.

Variable	Model I		Model II		Model III		Model IV	
	HR (95% CI)	*p*	HR (95% CI)	*p*	HR (95% CI)	*p*	HR (95% CI)	*p*
CAR	1.67 (1.43–1.94)	<0.001	1.33 (1.10–1.61)	0.003	1.33 (1.10–1.61)	0.003	1.30 (1.07–1.58)	0.009
Low CAR	Rf		Rf		Rf		Rf	
High CAR	2.50 (1.77–3.55)	<0.001	1.50 (1.04–2.18)	0.032	1.49 (1.03–2.17)	0.035	1.49 (1.02–2.17)	0.039

Model I adjusted for nothing. Model II adjusted for age, gender and BMI. Model III adjusted for model II plus ASA physical status, time to admission, cerebrovascular disease, lung disease and cancer. Model IV adjusted for Model III plus LDH, INR, hemoglobin, lymphocyte count and neutrophil count. CAR, creatinine-albumin ration; BMI, body mass index; ASA, American Society of Anesthesiologists; LDH, lactate dehydrogenase; INR, international normalized ratio; HR, hazard ratio; CI, confidence interval; Rf, reference.

### 3.3 Subgroup analysis

**Figure 4:**
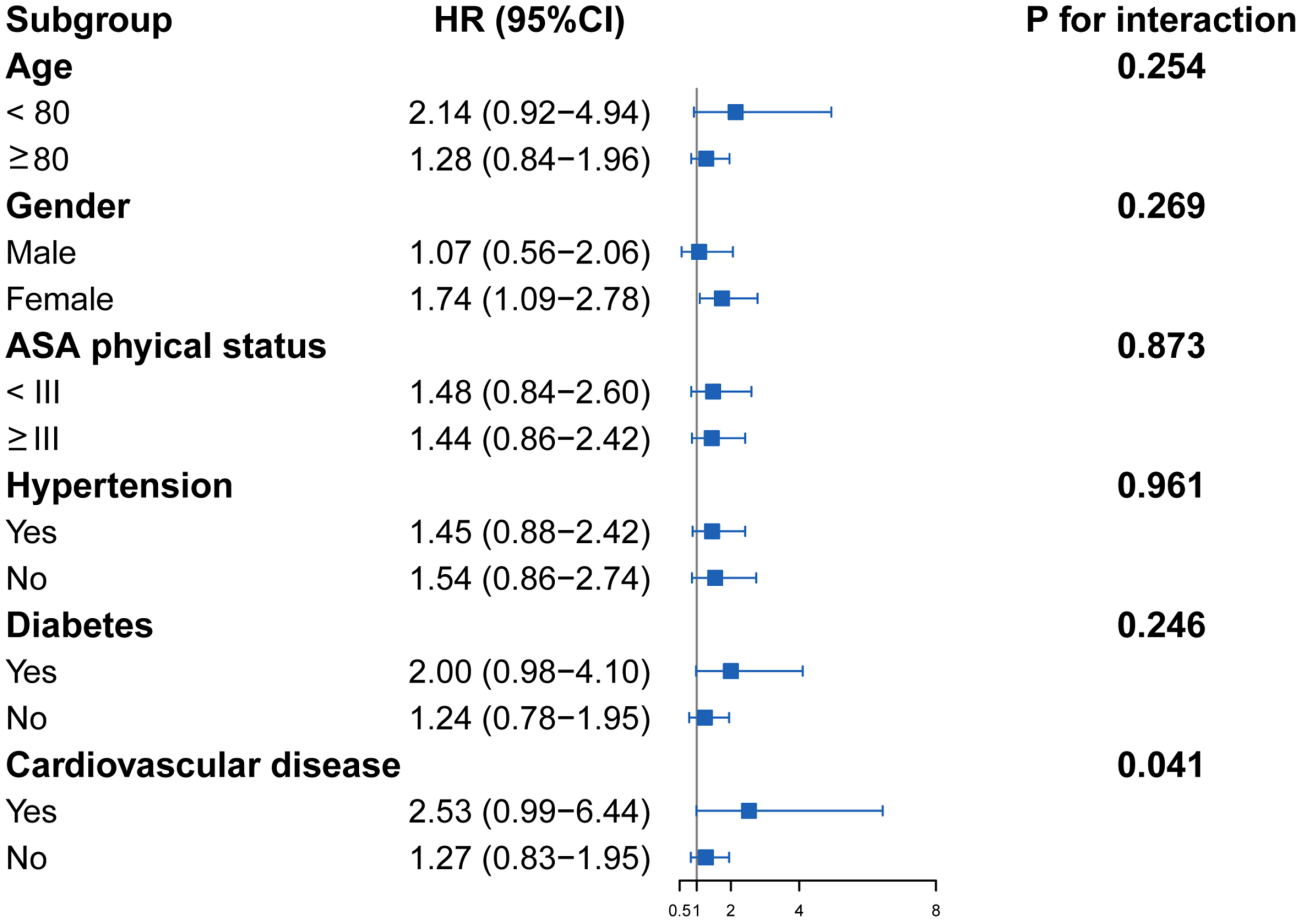
Forest plots of subgroup analysis of the relationship between CAR and 1-year mortality in patients with hip fracture surgery. Low CAR was the reference for high CAR. Each stratification adjusted for the factors (age, gender, BMI, ASA physical status, time to admission, cerebrovascular disease, lung disease, cancer, LDH, INR, hemoglobin, lymphocyte count, and neutrophil count) in the multivariable Cox regression, except for the stratification factor itself. BMI, body mass index; ASA, American Society of Anesthesiologists; LDH, lactate dehydrogenase; INR, international normalized ratio; CAR, creatinine-to-albumin ratio; HR, hazard ratio; CI, confidence interval.

Subgroup analysis showed that there was an interaction (*p* for interaction = 0.041) between cardiovascular disease and CAR on 1-year mortality. In addition, patients with high CAR generally exhibited a higher risk of 1-year mortality than those who had low CAR in all subgroups. Moreover, the association remained significant (HR = 1.74, 95% CI = 1.09–2.78) in female patients with hip fracture (Figure 4).

## 4 Discussion

This retrospective study included 1,770 older patients who underwent hip fracture surgery, with a 1-year mortality rate after surgery of 8.6%. Kaplan–Meier survival analysis indicated that patients with low CAR exhibited a high 1-year survival rate after surgery. In addition, multivariate Cox regression analysis revealed that high level of CAR was independently associated with increased 1-year mortality after hip fracture surgery. Furthermore, we found that the association remained consistent across all subgroups.

Increasing evidence shows that advanced age, low BMI, comorbidities, and prolonged time from injury to admission, have been identified as significant risk factors for increased mortality in patients with hip fracture (16–19). In addition, previous evidence has shown that peripheral blood inflammatory markers and nutritional indicators are related to prognosis in many clinical settings (20, 21). This is consistent with our findings. Moreover, we found that patients with high CAR had a high 1-year mortality rate after surgery.

Serum creatinine was used as a predictor for renal clearance capacity, which was associated with short- and long-term morbidity and mortality in patients with critical illness and cardiovascular disease (22, 23). Previous study has shown that elevated creatinine was associated with all-cause, cardiovascular and cancer mortality of American adults in a nationwide cohort study (24). In addition, a retrospective cohort study indicated that high level of creatinine was associated with an increased risk of mortality in patients undergoing surgery (25). Consistent with previous studies, we found that patients with 1-year mortality after surgery had a high level of serum creatinine.

Albumin is an important serum protein and has a wide range of physiological functions, such as immune regulation, endothelial stabilization, antioxidant effects (26–28). Besides, albumin can also be used as a biomarker for prognosis in many diseases, including inflammatory diseases, cancer, and hip fracture (29-31). Recent study showed that hypoalbuminemia was linked to poor postoperative recovery and increased mortality (32), which was similar with our study.

The CAR, as a comprehensive indicator, can more comprehensively reflect the metabolism, inflammation and nutritional status of patients, and is more closely related to the pathological and physiological characteristics in various clinical settings. Growing studies have reported that high level of CAR was linked to poor outcomes in patients with sepsis and acute pancreatitis (33, 34). In our study, we demonstrated the association of high CAR with increased 1-year mortality after hip fracture surgery in older patients. However, the definite causes and pathophysiological mechanisms underlying the relationship between CAR and a poor prognosis remain unclear. High CAR signifies a baseline state of elevated inflammation, coupled with probable muscle wasting and nutritional deficit, which cumulatively contribute to frailty and reduced physiological reserve after surgery in older patients with hip fracture.

We also found an interaction between cardiovascular disease and CAR on 1-year mortality. The association appeared substantially stronger in patients with cardiovascular disease (HR = 2.53), although the estimate was imprecise with a wide confidence interval (0.99–6.44). This imprecision is likely due to the limited sample size within the cardiovascular disease stratum. Biologically, this interaction may indicate that the inflammatory burden reflected by CAR synergizes with the compromised cardiovascular reserve in patients with cardiovascular disease, leading to a higher risk of mortality.

This study had several strengths. To our knowledge, it is the first large-scale retrospective study to examine the association between CAR and 1-year mortality in older patients undergoing hip fracture surgery. Second, the robustness of our findings was enhanced by comprehensive multivariable adjustments for potential confounders, minimizing selection bias and residual confounding. Third, subgroup analyses confirmed the consistency of our primary findings across different patient subgroups.

However, this study also had limitations. First, as a single-center retrospective study, the generalizability and causal relationship of our findings may be constrained. Second, despite rigorous statistical adjustments, unmeasured confounders such as variations in clinical care, postoperative complications, and socioeconomic factors could have influenced the observed association. Third, we excluded the patients with eGFR<60 mL/(min × 1.73 m^2^), limiting the generalizability of findings to patients with moderate-to-severe pre-existing kidney disease. Finally, the CAR levels were measured preoperatively but not tracked postoperatively, limiting insights into dynamic changes influencing outcomes. Therefore, multicenter studies with larger cohorts are needed to conform the association.

## 5 Conclusion

Our study demonstrated that high CAR was associated with increased 1-year mortality in older patients undergoing hip fracture surgery. Preoperative CAR could be a simple and effective strategy to flag patients at high risk for complications and poor recovery after surgery. However, multicenter, prospective studies are needed to validate these findings.

## Data Availability

The raw data supporting the conclusions of this article will be made available by the authors, without undue reservation.

## Footnotes

1
http://www.R-project.org
